# The clinical significance of tumor infiltrating lymphoctyes in breast cancer: does subtype matter?

**DOI:** 10.1186/1471-2407-12-135

**Published:** 2012-04-03

**Authors:** Jonathan Krell, Adam E Frampton, Justin Stebbing

**Affiliations:** 1 Department of Surgery and Cancer, Imperial College, Hammersmith Hospital, Du Cane Road, London W12 ONN, UK

**Keywords:** Tumor infiltrating lymphocytes, Cancer, Immunobiology

## Abstract

Tumor infiltrating lymphocytes (TILs) are commonly detected in breast tumors but their bearing on disease outcome is uncertain. The importance of TILs appears to be subtype-specific and varies depending on the histologic characteristics of the tumor. As our understanding of tumorigenesis is increasing the relevance of immunobiology will become apparent.

## Background

Breast cancer comprises many different subtypes characterized by distinguishing factors such as cell type of origin, grade and molecular specificities such as Ki-67 levels and receptor status. In recent years further molecular characteristics such as specific protein and miRNA expression levels have been associated with tumor phenotype [1,2], and it appears that multiple factors determine prognosis in this disease. Although a greater understanding of cancer cell biology is imperative to improving treatment, it has been shown that variability in the tumor microenvironment can also impact on outcome. The past 30 years have accumulated considerable evidence that tumors can elicit a significant immune response, and the body's normal defence mechanisms can play a key role in promoting or preventing carcinogenesis and tumor spread [3]. Tumors are commonly infiltrated by lymphocytes (TILs) and their number and subtype are considered to reflect the host immune response to malignancy [4]. The concept of cancer immunobiology appears to be an important one and the possible role of TILs in determining outcome in breast and other cancers needs further evaluation.

## Main text

Cytotoxic CD8^+ ^and CD4^+ ^Th1 T cells function as the major anti-tumor lymphocytes predominantly through the production of interferon-gamma (IFN-γ), whereas interleukin-6 (IL-6), tumor-necrosis factor (TNF) and IL-23 produced by tumor-associated macrophages (TAM) or myeloid-derived suppressive cells (MDSC) are believed to promote tumor formation and growth. The roles played by TILs such as CD4^+ ^CD25^+ ^Foxp3^+ ^TILs (Tregs) and Th17 cells remains less clear but they appear to suppress the activity of effector cells including CD4+ and CD8+ cytotoxic T cells, natural killer cells, natural killer T cells, and B cells [5,6]. This activity may promote the survival of cancer cells by affording protection from both the innate and adaptive immune systems. Several studies have shown that tumor infiltration by effector T lymphocytes is associated with favorable prognosis [7,8], but that higher numbers of Tregs are associated with progression in a variety of malignancies [9,10].

The clinical significance of TILs in breast cancer remains controversial and lymphocyte location and subtype appears to determine outcome. High CD4+ and CD8+ lymphocytic infiltration has been associated with positive lymph node status as well as worse overall survival [11], but higher relative levels of CD8+ TILs in ER negative breast cancer correlates with a better prognosis [12]. Infiltration by Tregs correlates with tumor invasiveness and was shown to represent an independent unfavorable prognostic factor, especially in lymph node positive breast cancer [13]. TIL levels have also been associated with response to breast cancer therapy. Treg levels were significantly reduced during treatment with trastuzumab whilst Th17 frequencies were concomitantly increased [14] and TIL counts have been shown to represent an independent predictor of response to neo-adjuvant paclitaxel [15].

The paper by Droeser and coworkers in this addition of BMC cancer addresses the role of TILs in breast cancer and particularly focuses on the differential lymphocyte infiltration between histological subtypes. Furthermore, they utilise an extensive clinical follow-up database to correlate TIL number in different tumor compartments with clinico-pathological features and outcome data. The study is significant in a number of ways: Firstly, despite being a retrospective study the sample size is large and certainly sufficient for the purpose of the analysis. Secondly it addresses issues that have not previously been reported such as T-cell infiltration in different histological subtypes and the occurrence of IL-17+ lymphocytes in breast cancer tissue. They used standard immunohistochemical techniques to stain for TILs in a tissue microarray that included 894 ductal and 164 lobular breast cancers and correlated lymphocyte counts with clinico-pathological parameters and survival. The major findings were that ductal and lobular breast cancers appear to be infiltrated by different lymphocyte subpopulations and that in ductal cancers increased CD4+ and FOXP3+ lymphocyte infiltration was linked to more aggressive histological features (such as higher grade and ER-negative status) but in lobular carcinomas lymphocyte infiltration was not linked to any clinico-pathological parameters. Interestingly, although no prognostic relevance could be attributed to absolute TIL number in either histological subtype, a FOXP3+ to CD4+ ratio of greater than one in ductal carcinoma seemed to represent an independent favorable prognostic variable. IL-17 lymphocyte numbers were low in both histological subtypes and no significant difference between TILs was described between tumor or stroma tissue.

## Discussion

So what is the true clinical relevance of the current study by Droeser *et al.*? It provides further evidence that evaluating Treg tumor infiltration may be a useful adjunct to current methods used for staging breast cancer and may serve as a reliable prognostic biomarker. However the data suggest that it is the relative infiltration of different Tregs rather than absolute number that is most important in this respect. Furthermore, similar future studies in the metastatic setting may demonstrate a role for TILs in predicting the behavior of advanced tumors. The study also suggests a possible role for TILs as diagnostic biomarkers as there is a clear difference in lymphoctye infiltration between histological subgroups. This finding may also provide valuable information as to how variability in immunogenicity is associated with different tumors or tumor growth patterns. A greater understanding of the function of TILs within a tumor might also aid in identifying those patients most likely to benefit from immunomodulatory therapies in breast cancer, a strategy that has been used with varying success in other tumor types such as melanoma [16] and renal cell carcinoma [17].

## Conclusion

In an era when standard prognostic, predictive and diagnostic parameters are ever changing and the use of extended molecular fingerprint analyses are increasing, we are continually looking for techniques to further our understanding of tumor biology and improve patient outcome. Although further work is clearly required to fully establish the importance and potential role of TILs in breast and other malignancies, it seems clear that a thorough assessment of the immunobiological status of tumors will go someway to achieving these goals. The relevance of tumor lymphocyte infiltration cannot be ignored but needs to be properly evaluated in larger prospective studies which must encompass the parameters set out in this and previous studies.

## Competing interests

The authors declare that they have no competing interests.

## Authors' contributions

JK prepared the main text, discussion and conclusion. AEF prepared the background and edited the manuscript. JS supervised the writing of the manuscript and edited the draft.

## Pre-publication history

The pre-publication history for this paper can be accessed here:

http://www.biomedcentral.com/1471-2407/12/135/prepub
